# Successful cardiac synchronization therapy device upgrade using an active fixation quadripolar pacing lead in a patients with persistent left superior vena cava and absent right superior vena cava

**DOI:** 10.1002/joa3.12905

**Published:** 2023-07-31

**Authors:** Shushi Nishiwaki, Satoshi Shizuta, Munekazu Tanaka, Hirohiko Kohjitani, Koh Ono

**Affiliations:** ^1^ Department of Cardiovascular Medicine Kyoto University Graduate School of Medicine Kyoto Japan

**Keywords:** absent right superior vena cava, active fixation lead, cardiac resynchronization therapy, pacing‐induced cardiomyopathy, persistent left superior vena cava

Persistent left superior vena cava (PLSVC) is a common congenital anomaly, with an incidence of 0.5%.^1^ Although PLSVC is usually asymptomatic, cardiac implantable electrical devices (CIEDs) should be implanted through the right superior vena cava from the right side because implantation through the PLSVC is technically difficult. However, in patients with PLSVC concomitant with an absent right superior vena cava (ARSVC), CIEDs must be implanted through the PLSVC. Several case reports of cardiac resynchronization therapy (CRT) device implantation through the PLSVC have been published, but left ventricular (LV) lead placement remains challenging in these patients.^2^, ^3^, ^4^, ^5^ Here, we report a successful cardiac synchronization therapy device upgrade using an active fixation quadripolar pacing lead in a patient with PLSVC and ARSVC.

The patient, a 70‐year‐old man, had visited our outpatient clinic 8 years prior, after experiencing dyspnea. A 12‐lead electrocardiogram (ECG) showed complete atrioventricular block with an escape rhythm of 43 bpm. Transthoracic echocardiography (TTE) showed a left ventricular ejection fraction (LVEF) of 58%. We attempted to insert a temporary pacing lead from the right internal jugular vein; however, the guidewire did not progress directly to the heart. Venography revealed PLSVC and ARSVC (Figure 1A). A temporary pacing lead was inserted into the right ventricle through the right femoral vein. We implanted a dual‐chamber pacemaker (PM) (PM generator: Reply DR; Sorin, atrial/ventricular lead: SelectSecure 3830; Medtronic) from the left side using a deflectable guiding sheath (SelectSite C304; Medtronic) (Figure 1B). Five years after PM implantation, he developed dyspnea; TTE revealed a deterioration of LVEF to 33%. The ventricular pacing rate was 100%, with a wide QRS duration of 174 ms (Figure 1C). TTE revealed septal flash and intraventricular dyssynchrony. As no other cause of LVEF deterioration was identified, the patient was diagnosed with pacing‐induced cardiomyopathy. After starting optimal medical therapy, the LVEF slightly improved to 41% but did not normalize.

**FIGURE 1:**
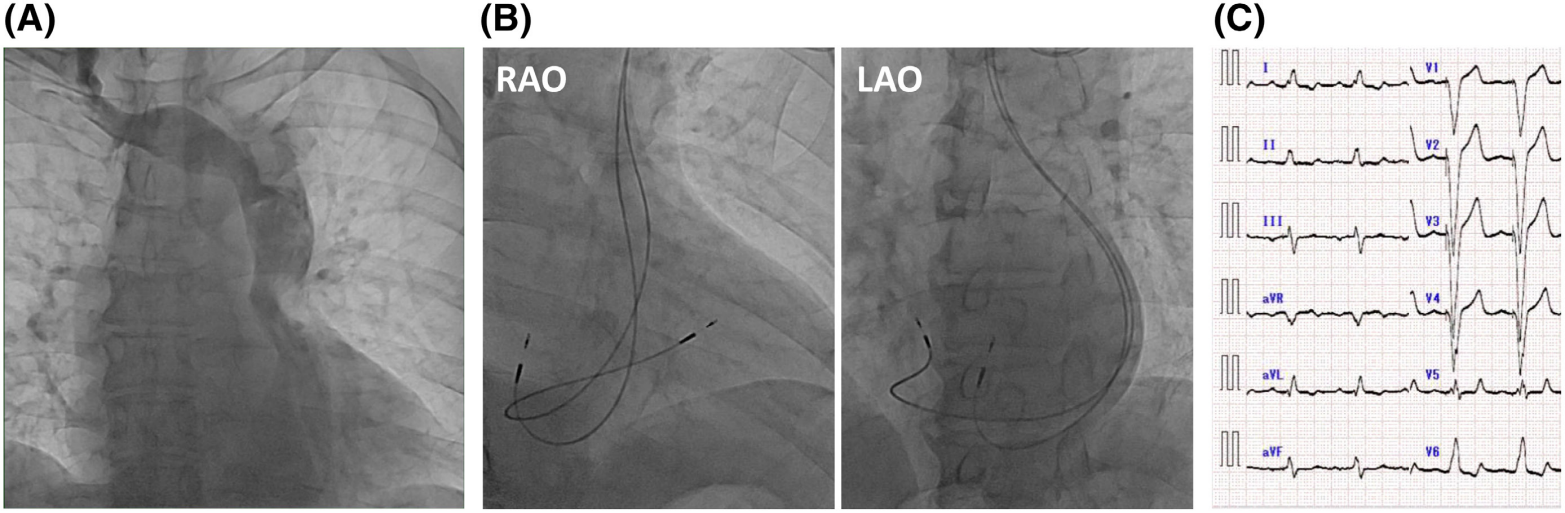
(A) Venography revealed PLSVC and ARSVC. (B) Chest x‐ray after initial pacemaker implantation. (C) Twelve‐lead ECG after pacemaker implantation. QRS duration was 174 ms. ARSVC, absent right superior vena cava; ECG, electrocardiogram; PLSVC, persistent vena cava.

Eight years after PM implantation, he was admitted to our hospital for battery exchange. Since he still felt dyspnea while walking, we decided to perform a CRT device upgrade concurrent with the battery exchange. Preoperative three‐dimensional images of the PLSVC and coronary sinus (CS) were obtained using ECG‐gated contrast‐enhanced computed tomography (CECT) (Figure 2A), and the posterolateral and lateral veins were identified. The right ventricular lead had been placed in the right ventricular outflow tract, opposite the posterolateral vein. Although the posterolateral vein seemed to drain near the junction between the PLSVC and the CS, the precise site was ambiguous. We performed coronary angiography to confirm the relationship between the PLSVC and the CS (Figure 2B). Contrast jet flow from the CS to the PLSVC clarified that the posterolateral vein was very close to the junction between the PLSVC and the CS. Therefore, we decided to target the posterolateral vein.

**FIGURE 2:**
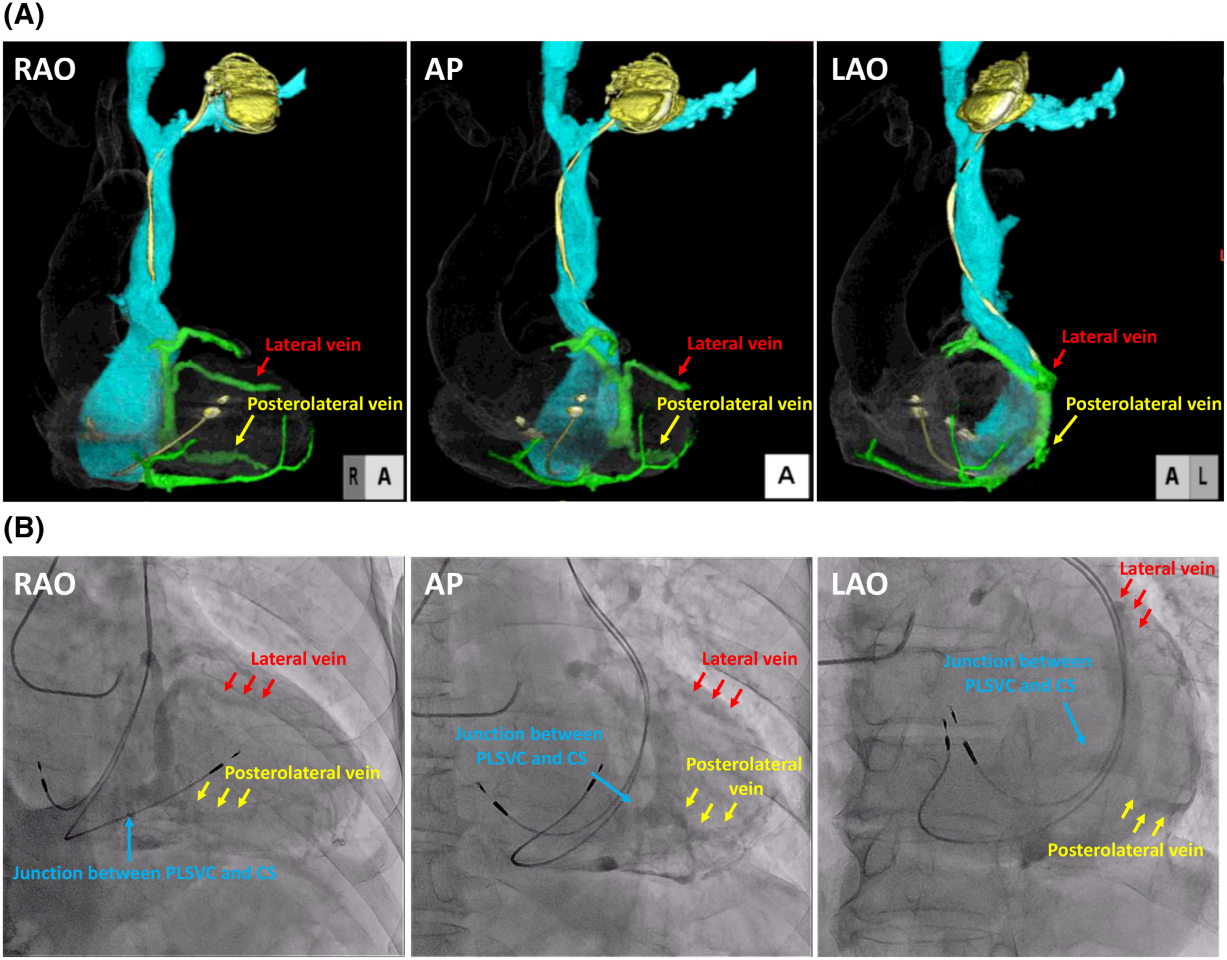
(A) Three‐dimensional images of the PLSVC and the CS were obtained using ECG‐gated contrast‐enhanced computed tomography. (B) Delay phase of coronary angiography. Contrast jet flow clarified the junction between the PLSVC and the CS. AP, anterior posterior; CS, coronary sinus; ECG, electrocardiogram; LAO, left anterior oblique; PLSVC, persistent left superior vena cava; RAO, right anterior oblique.

The subclavian vein was punctured and a 0.035 inch hydrophilic guidewire was advanced into the right atrium through the PLSVC. A 9Fr introducer sheath (MediKit) was inserted into the PLSVC. A delivery catheter with 130° curve (Attain Select II; Medtronic) was advanced to the junction between the PLSVC and the CS and contrast agent was injected, revealing the posterolateral vein (Figure 3A). A 0.014 inch wire (Attain Hybrid; Medtronic) was inserted into the posterolateral vein (Figure 3B). An active fixation quadripolar lead (Attain Stability Quad; Medtronic) was advanced over the wire into the posterolateral vein (Figure 3C). Because the parameters at LV 2–4 were acceptable (threshold: 1.5 V/0.4 ms; impedance: 608 Ω; no phrenic nerve stimulation at 8 V/0.4 ms), the quadripolar lead was fixed using a side helix. After removing the guiding catheter and sheath, the existing right atrium/ventricular leads and the new LV lead were connected to a CRT generator (Percepta Quad CRT‐P; Medtronic). The chest x‐ray on the following day showed no dislodgement of the LV lead (Figure 4A). In the 12‐lead ECG, the QRS duration of biventricular pacing narrowed from 170 to 140 ms (Figure 4B). One year after the upgrade, TTE revealed LVEF had improved to 63%, and dyspnea was relieved.

**FIGURE 3:**
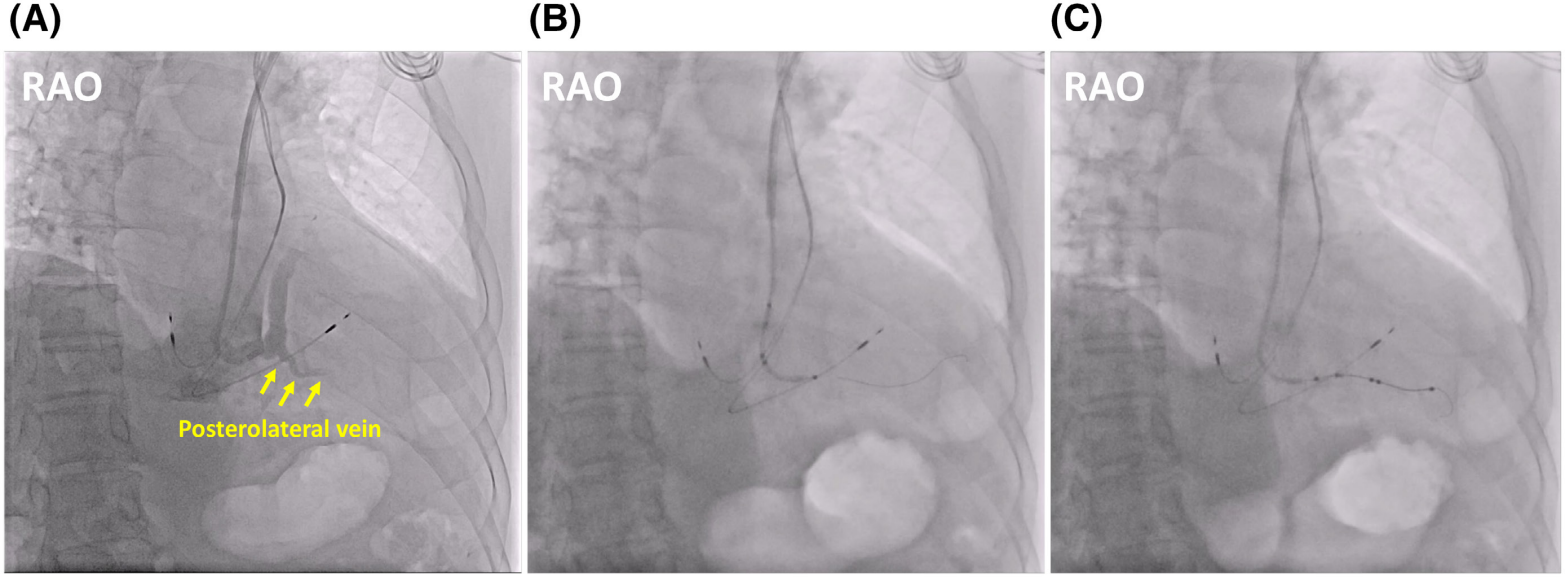
(A) Contrast agent was injected from the delivery catheter, revealing the posterolateral vein. (B) A 0.014 inch wire was inserted into the posterolateral vein. (C) An active fixation quadripolar lead was advanced over the wire into the posterolateral vein. RAO, right anterior oblique.

**FIGURE 4:**
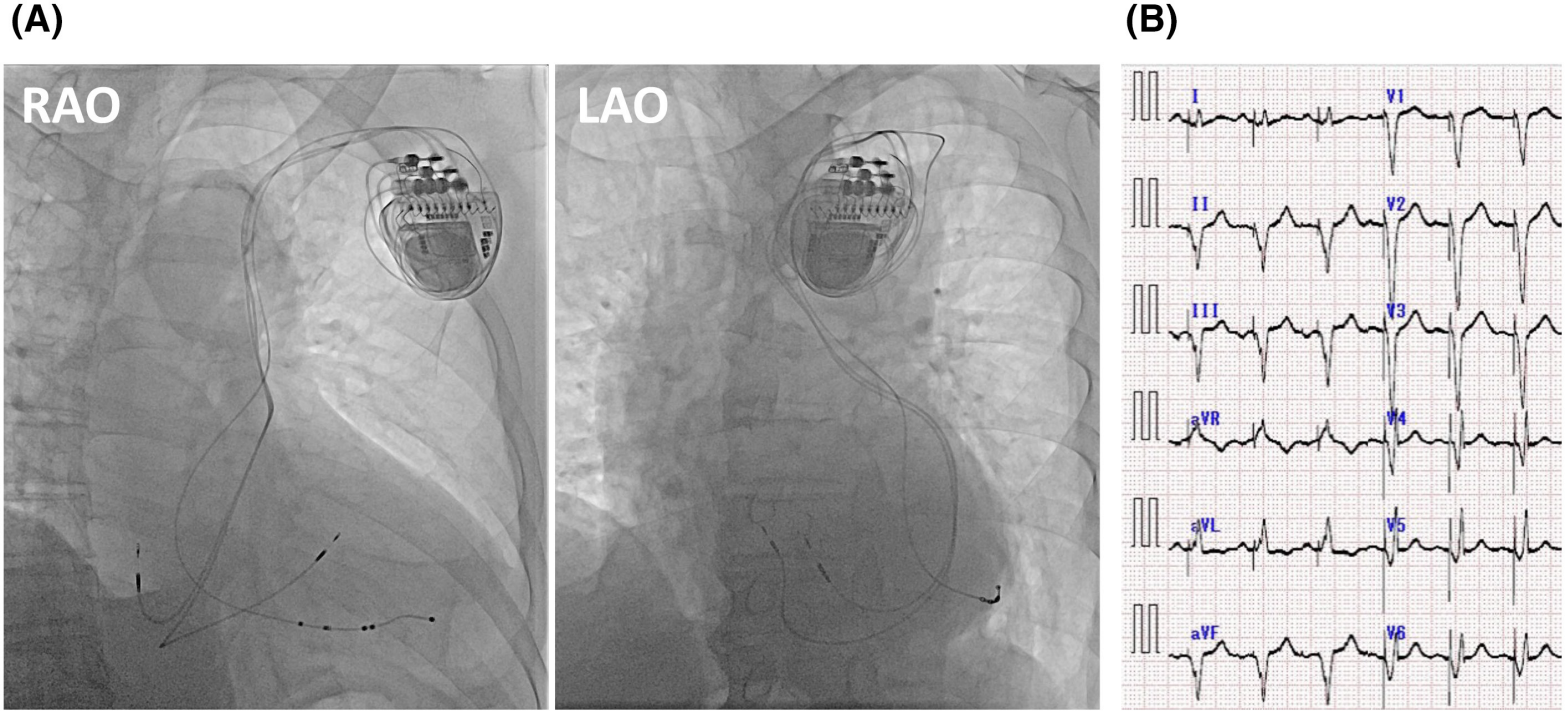
(A) Chest x‐ray after CRT device upgrade. (B) Twelve‐lead ECG after CRT device upgrade. Biventricular pacing shortened QRS duration from 170 to 140 ms. CRT, cardiac resynchronization therapy; ECG, electrocardiogram.

In patients with PLSVC and ARSVC, it is difficult to identify all branches of the CS during the CRT device upgrade procedure because retrograde venography using a balloon occlusion catheter is usually impossible. Because 90% of the PLSVC drains into the CS, the LV leads pass through in the following order: PLSVC, junction between PLSVC and CS, main body of the CS, and branch of the CS.^1^ In a previous report, three‐dimensional CECT were used only to detect the CS branches.^4^ However, the difficulty of the procedure is likely to depend more on the relationship between the junction site and the CS branches than on the number or location of the CS branches. In our case, although the precise site of the PLSVC/CS junction remained ambiguous after three‐dimensional CECT, contrast jet flow from the CS to the PLSVC in delayed coronary angiography images clarified the junction position. Moreover, the posterolateral vein is close to the junction site. Therefore, preoperative CECT and coronary angiography are both important for a success procedure.

Sufficient LV lead slack is usually left in the right atrium to prevent LV lead dislodgement during CRT device implantation. However, patients with PLSVC and ARSVC have insufficient space to leave the LV lead because it does not pass through the right atrium. The active fixation lead can be fixed using side helix, decreasing the risk of LV lead dislodgement, even in patients with PLSVC and ARSVC.^3^, ^4^, ^5^ In addition, the active fixation quadripolar lead can be fixed at any position of the CS branch and also has four electrodes, which increase the probability of LV lead placement in an easily accessible branch. As the long‐term outcome of lead extraction is unknown for active fixation leads, we hesitate to use the lead in all CRT cases. However, the active fixation leads are a good option for complicated cases with limited accessible branches, such as PLSVC/ARSVC.

## FUNDING INFORMATION

None.

## CONFLICT OF INTEREST STATEMENT

The authors declared that no competing interests exist.

## PATIENT CONSENT STATEMENT

The patient provided consent for publication.
